# Bone marrow dosimetry in low volume mHSPC patients receiving Lu-177-PSMA therapy using SPECT/CT

**DOI:** 10.1186/s40658-024-00636-0

**Published:** 2024-04-03

**Authors:** Dagmar Grob, Bastiaan M. Privé, Constantijn H. J. Muselaers, Niven Mehra, James Nagarajah, Mark W. Konijnenberg, Steffie M. B. Peters

**Affiliations:** 1 Department of Medical Imaging, Radboud University Medical Center, P.O. Box 9101, 6500 HB Nijmegen, The Netherlands; 2 Department of Healthcare and Information Technology, Slingeland Hospital, Doetinchem, The Netherlands; 3 Department of Radiation Oncology, Erasmus Medical Center, Rotterdam, The Netherlands; 4 Department of Radiology and Nuclear Medicine, Erasmus Medical Center, Rotterdam, The Netherlands; 5 Department of Urology, Radboud University Medical Center, Nijmegen, The Netherlands; 6 Department of Medical Oncology, Radboud University Medical Center, Nijmegen, The Netherlands

**Keywords:** Bone marrow dosimetry, Absorbed dose, Radionuclide therapy, 177-Lu-PSMA

## Abstract

**Background:**

Bone marrow toxicity in advanced prostate cancer patients who receive [^177^Lu]Lu-PSMA-617 is a well-known concern. In early stage patients; e.g. low volume metastatic hormone sensitive prostate cancer (mHSPC) patients, prevention of late bone marrow toxicity is even more crucial due to longer life expectancy. To date, bone marrow dosimetry is primarily performed using blood sampling. This method is time consuming and does not account for possible active bone marrow uptake. Therefore other methodologies are investigated. We calculated the bone marrow absorbed dose for [^177^Lu]Lu-PSMA-617 in mHSPC patients using SPECT/CT imaging and compared it to the blood sampling method as reference.

**Methods:**

Eight mHSPC patients underwent two cycles (3 and 6 GBq) of [^177^Lu]Lu-PSMA-617 therapy. After each cycle, five time point (1 h, 1 day, 2 days, 3 days, 7 days) SPECT/CT was performed at kidney level. Bone marrow dosimetry was performed using commercial software by drawing ten 1.5 cm diameter spheres in the lowest ten vertebrae to determine the time-integrated activity. Simplified protocols using only 2 imaging time points and 3 vertebrae were also compared. Blood-based dosimetry was based on the blood sampling method according to the EANM guideline.

**Results:**

Mean bone marrow absorbed dose was significantly different (*p* < 0.01) for the imaging based method (25.4 ± 8.7 mGy/GBq) and the blood based method (17.2 ± 3.4 mGy/GBq), with an increasing absorbed dose ratio between both methods over time. Bland Altman analysis of both simplification steps showed that differences in absorbed dose were all within the 95% limits of agreement.

**Conclusion:**

This study showed that bone marrow absorbed dose after [^177^Lu]Lu-PSMA-617 can be determined using an imaging-based method of the lower vertebrae, and simplified using 2 time points (1 and 7 days) and 3 vertebrae. An increasing absorbed dose ratio over time between the imaging-based method and blood-based method suggests that there might be specific bone marrow binding of [^177^Lu]Lu-PSMA-617.

**Supplementary Information:**

The online version contains supplementary material available at 10.1186/s40658-024-00636-0.

## Background

Radionuclide therapy using [^177^Lu]Lu-PSMA-617 has been increasingly applied in patients with metastasized castrate resistant prostate cancer (CRPC) [1–5], showing improved progression free and overall survival. Following the phase III Vision trial [4], the compound was granted both FDA and EMA approval in 2022. With prostate cancer being one of the most common non-skin cancers worldwide [6], a large number of patients will be eligible for this treatment. The most common adverse events reported were fatigue, (mild) dry mouth and nausea, all grade I–II [4]. However, the most important concern for this treatment is the risk on grade III–IV bone marrow toxicity, with the incidence of thrombocytopenia and lymphopenia considerably higher in the treated group (8%) compared to the control group (≤ 1%). Especially in these heavily pre-treated patients, bone marrow capacity might be compromised.

What’s more, patients in earlier disease stage (e.g. still in a hormone sensitive setting) could potentially also benefit from treatment with [^177^Lu]Lu-PSMA-617 [7, 8]. In these patient cohorts, prevention of bone marrow toxicity is even more crucial due to the long life expectancy and other available treatment options.

For this reason, performing reliable bone marrow dosimetry in patients receiving [^177^Lu]Lu-PSMA-617 therapy is of great interest. Following the EANM guidelines on bone marrow dosimetry [9], measuring the activity in plasma by means of blood sampling is considered the gold standard for treatments without active uptake in bone or bone marrow, as is assumed for [^177^Lu]Lu-PSMA-617 [10–13]. However, some studies looking into bone marrow dosimetry for other compounds suggest that using plasma activity for bone marrow dosimetry might not be suitable to predict the haematological toxicity, while imaging-based dosimetry might be a better predictor. For example in ^90^Y-antibody treatment of Non-Hodgkin’s lymphoma it was shown that blood-based bone marrow absorbed dose did not correlate with bone marrow toxicity [14] while lumbar vertebrae imaging dosimetry did [15]. Similarly, in [^177^Lu]Lu-DOTATATE for treatment of neuroendocrine tumors, imaging based dosimetry was shown to better correlate with bone marrow toxicity [16–18] than the blood based method.

Moreover, blood sampling is considered a time-consuming methodology, whereas post-treatment imaging is usually performed in some form anyway, so these scans are generally available to use for dosimetry.

The goal of this study was to investigate the possibility to use post-treatment SPECT imaging for bone marrow dosimetry in [^177^Lu]Lu-PSMA-617 therapy, and to evaluate possible deviation of absorbed dose estimates compared to the blood sampling method. We retrospectively used data from low volume metastasized hormone sensitive prostate cancer (mHSPC) patients, who all had limited bone lesion burden and received both 5-time-point SPECT/CT imaging and 9-time-point blood sampling post-treatment.

Furthermore, for clinical implementation, we investigated the possibility to limit the number of time points for bone marrow dosimetry. For other organs at risk (kidneys, salivary glands) and lesions it has been suggested that one or two imaging time points are sufficient to reliably calculate the absorbed dose [19–21]. Therefore, it would be practically convenient if these images could also be used to calculate the bone marrow absorbed dose.

## Methods

### Study design and patient population

The data set comprised of imaging and blood data of 8 patients with low volume mHSPC who received [^177^Lu]Lu-PSMA therapy. The initial prospective study was approved by the Medical Review Ethics Committee Region Arnhem–Nijmegen and was registered on clinicaltrials.gov (NCT03828838). All subjects signed an informed consent form. A comprehensive description of the patient population and clinical results has been published earlier [7]. In short, mHSPC patients with prostate-specific antigen (PSA) doubling time ≤ 6 months and ≤ 10 visible metastases on baseline [^68^Ga]Ga-PSMA-PET/CT, with at least one lesion ≥ 10 mm in diameter, were included. All patients underwent two cycles of [^177^Lu]Lu-PSMA therapy (cycle 1: 3.1 ± 0.1 GBq, cycle 2: 5.9 ± 0.4 GBq). See Additional file 1: Online Resource 1 for the study flowchart.

### Bone marrow dosimetry using blood sampling

Volumetric organ based dosimetry was performed according to the scheme defined by the Committee on Medical Internal Radiation Dose (MIRD) [22], calculating the absorbed dose using the MIRD equation:

1 $$D\left( {r_{T} } \right) = \mathop \sum \limits_{{r_{S} }} \tilde{A}\left( {r_{S} } \right) \times S\left( {r_{T} \leftarrow r_{S} } \right)$$

where *D* is de absorbed dose (mGy), ${\tilde{\text{A}}}$ is the time integrated activity (MBq.h) and S is the ‘S-value’ (mGy/MBq.h); the absorbed dose rate in target organ r_T_ per unit activity in source organ r_S_.

In the previous study, bone marrow dosimetry using blood sampling was described for this patient population [23]. In short, after each therapy, blood draws were collected at 5, 30, 60, 120 and 180 min and 1, 2, 3 and 7 days post injection (p.i.). Blood samples were measured in a scintillation counter (248 WIZARD^2^, Perkin Elmer, Groningen, The Netherlands) that was calibrated for ^177^Lu to translate from counts per minute (CPM) to megabecquerels (MBq) per volume unit (ml). Time-activity curves were fitted to a three-exponential decay using GraphPad Prism 5.03 (Graphpad Software Inc., CA, USA). In the blood-based method the ratio of activity concentration in blood to that in bone marrow was assumed to be 1 for [^177^Lu]Lu-PSMA.

### Bone marrow dosimetry using SPECT/CT imaging

After each therapy, SPECT/CT imaging of the pelvic/kidney region was performed at 1 h and 1, 2, 3, and 7 days p.i. on either a Symbia T16 or Symbia Intevo Bold system (Siemens Healthineers, Erlangen, Germany). Acquisition and reconstruction parameters can be found in Additional file 1: Online Resource 2. None of the patients had any bone lesions in the region of interest.

**Fig. 1:**
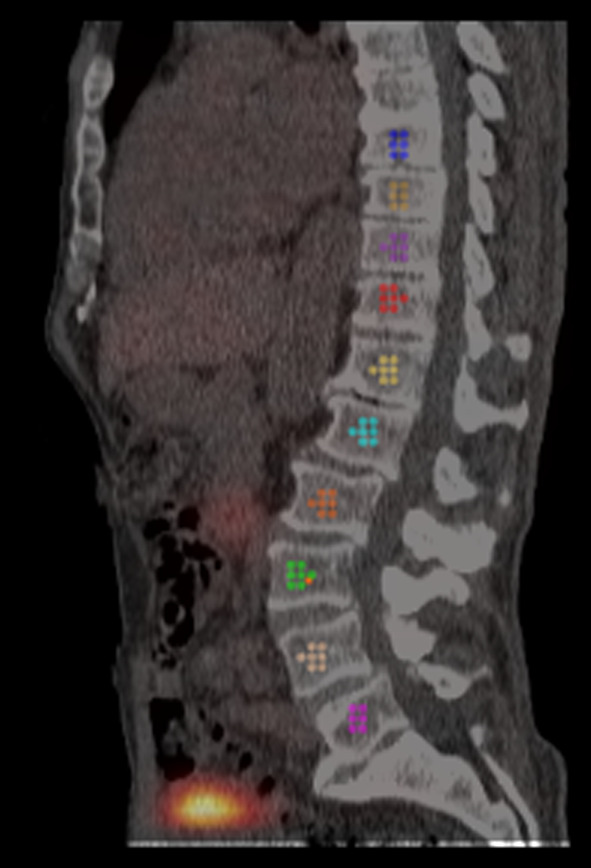
Illustration of VOI drawing in vertebrae L1-L5 and T8-T12. All VOIs were spherical with 1.5 cm diameter

Since about 28% of active red marrow is located in the thoracic and lumbar spine according to International Commission on Radiological Protection (ICRP) Publication 89 [24], all 5 lumbar vertebrae (L1–L5) and the lower 5 thoracic vertebrae (T8–T12) were used to draw CT-based 1.5 cm diameter spheric volumes of interest (VOIs) for dosimetry, using Hermes Dosimetry v2.15.0.81 (Hermes Medical Solutions, Sweden, Stockholm) as illustrated in Fig. 1. The total counts for the 10 vertebrae combined were determined for each time point and converted to Becquerels using the scanner specific calibration factor (cpm/ml per kBq/ml). The time integrated activity was determined in GraphPad Prism 9.5.0 using a one-phase decay fit, weighted to 1/SD^2^ and extrapolated to t = 0 h. To calculate total bone marrow absorbed dose, a vertebra density of 1.015 g/cm^3^ was used (ICRU report 46 [25]), taking 70% cellularity of each red marrow space within the vertebra to be hematopoietic according to ICRP 70 [26]. Furthermore, a total red bone marrow weight of 1170g was used according to the ICRP 89 adult male human model [24], with a corresponding S-value of 4.14 × 10^−2^ mGy/(MBq h). Since these patients did not receive any cancer related systemic therapies, these assumptions seem applicable.

### Protocol simplification

Depending on the SPECT bed position, not all 10 vertebrae used in the above method might be visible. Therefore, it would be desirable to use fewer vertebrae (e.g. three) if possible. To select the most appropriate vertebrae for further dosimetry, uptake distribution for the 5 lumbar vertebrae and 5 lower thoracic vertebrae was evaluated. In addition, it was considered that the selected vertebrae needed be visible on one standard bed position SPECT when dosimetry will be done in clinical setting.

In addition, imaging based absorbed dose calculations were performed using fewer time points. In a previous study on dosimetry for kidney, salivary glands and lesions it was shown that reliable absorbed dose calculations can be performed based on two time points; one early (1 or 2 days p.i.) and one late time point (7 days p.i.) [19]. Since this would therefore be a suitable protocol for clinical routine, it was evaluated whether bone marrow dosimetry based on a SPECT/CT scan at 24 h and 7 days would be comparable to 5-time-point dosimetry.

### Statistical analysis

Uncertainties in absorbed dose calculations were performed based on the EANM uncertainty guideline [27]. Overall, a systematic uncertainty arises in taking the red marrow distribution according to standard adult male human model (ICRP 89). The main contribution to the uncertainty in the imaging based dosimetry comes from the time-activity curve, obtained by single exponential fitting using Poisson error as weight and applying correction for covariance in its parameters. Other uncertainties were ignored, as they were comparable to what was used in the blood-based method, uncertainties for the blood based method have been described earlier [23]. An elaborate description of the uncertainty analysis can be found in Additional file 1: Online Resource 3. Differences between blood-based dosimetry and various imaging-based protocols were evaluated using the Wilcoxon matched-pair signed rank test (statistically different for *p* < 0.05) and Bland–Altman analysis.

## Results

A total of 14 therapy cycles could be analyzed, as for 2 cycles the SPECT/CT at 7 days was missing so no reliable uptake curve could be fitted. Of these 14 cycles, 6 had all 10 vertebrae (T8–L5) available for analysis, 5 were missing either T8 or L5 in the SPECT field of view (FOV), and 3 were missing both T8 and T9.

### Blood based dosimetry versus imaging based dosimetry

**Fig. 2:**
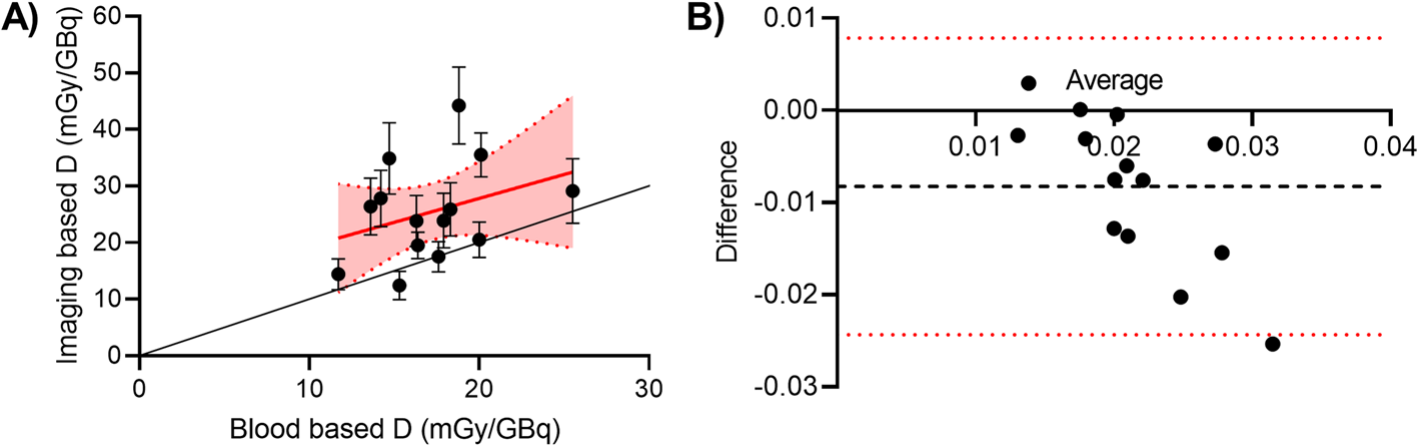
Comparison between bone marrow absorbed dose based on blood sampling and based on imaging data. **A** Imaging based dosimetry generally yields a slightly higher absorbed dose than blood based dosimetry. Each dot represents a therapy cycle, vertical bars represent uncertainty. Red line is the linear regression line including error bands. **B** Bland Altman analysis of difference (blood based absorbed dose minus imaging absorbed dose) versus average absorbed dose of the two methodologies. Black dotted line represents the bias (average of the differences), red dotted lines represent the 95% limits of agreement

**Fig. 3:**
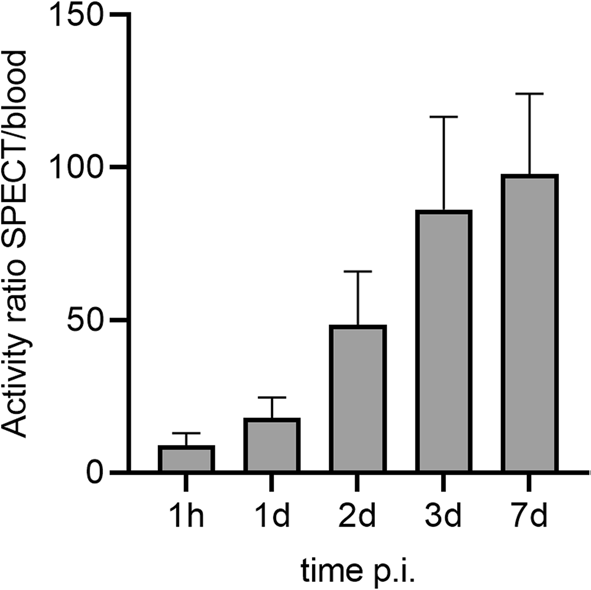
The ratio between activity measured on SPECT VOIs (10 vertebrae) and activity measured in blood samples, for the 5 different time points in this study

Mean bone marrow absorbed dose was significantly different (*p* < 0.01) for the imaging based method (25.4 ± 8.7 mGy/GBq with a mean error of 17.1%) and the blood based method (17.2 ± 3.4 mGy/GBq with mean error 31.1%). For an overview of all absorbed doses per cycle and methodology, including uncertainty, see Table 1. Figure 2A shows that the absorbed dose determined using imaging data was usually slightly higher, and that the variation between patients/therapy cycles was larger. The Bland Altman analysis in Fig. 2B shows that almost all difference values (blood based absorbed dose—imaging based absorbed dose) fall within the 95% limits of agreement. A comparison in activity uptake at specific time points between the imaging data and blood samples showed that the ratio between SPECT activity and blood activity increased for later time points (Fig. 3).

### Simplification of the imaging based dosimetry protocol

**Fig. 4:**
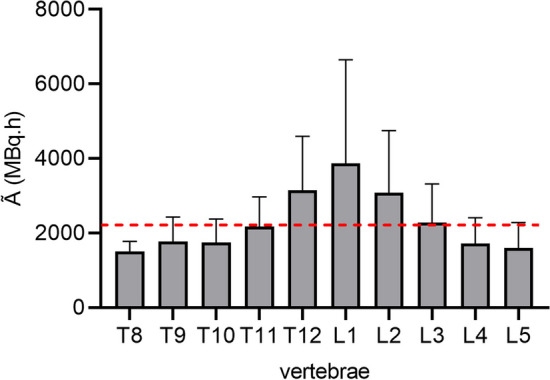
Distribution of time integrated activity for thoracic vertebra T8 to lumbar vertebra L5. Red dashed line represents the mean time integrated activity (2289 MBq.h)

**Fig. 5:**
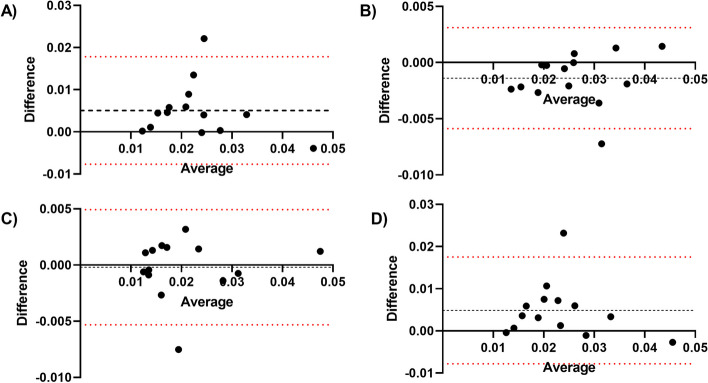
Bland Altman analysis for comparison of different imaging-based bone marrow dosimetry protocols. Black dotted lines represent the bias (average of the differences), red dotted lines represent the 95% limits of agreement. **A** Comparison between the use of 10 and 3 vertebrae for a 5-time point protocol; **B** Comparison between the use of a 5 time point protocol and 2 time point protocol using 10 vertebrae; **C** Comparison between the use of a 5 time point protocol and 2 time point protocol using 3 vertebrae; **D** Comparison between the use of a 5 time point protocol using 10 vertebrae (full protocol) and a 2 time point protocol using 3 vertebrae (simplified protocol)

To select the most suitable vertebrae for calculation of bone marrow dose, the time integrated activity ${\tilde{\text{A}}}$ (MBq.h) was calculated for thoracic vertebra T8 to lumbar vertebra L5 (Fig. 4). Mean total bone marrow ${\tilde{\text{A}}}$ was 2289 ± 1066 MBq h. Vertebrae T12 to L2 generally yield higher ${\tilde{\text{A}}}$. Considering that vertebrae L3–L5 have comparable uptake and are (almost) always visible on the standard bed position SPECT/CT, it was decided to evaluate a simplification of the absorbed dose calculation method based on these three vertebrae. The resulting mean bone marrow absorbed dose was 20.4 ± 9.9 mGy/GBq with a mean error of 18.5% (Table 1). Figure 5A shows the Bland Altman analysis of the bone marrow absorbed dose based on 10 vertebrae (T8–L5) versus based on 3 vertebrae (L3–L5), showing that indeed the absorbed dose based on L3–L5 is slightly lower, but again within the 95% limits of agreements for all cycles.

For simplification to fewer time points the bone marrow absorbed dose was calculated based on two imaging time points (1 and 7 days) and either 10 or 3 vertebrae. This yielded a mean absorbed dose of 26.8 ± 8.4 mGy/GBq with 23.3% mean error and 20.6 ± 9.7 mGy/GBq with 27.2% mean error for 10 and 3 vertebrae, respectively (Table 1). Bland Altman analysis of both simplification steps showed that differences in absorbed dose were all within the 95% limits of agreement with exception of one cycle (Figure 5B, C). Figure 5D shows the Bland Altman comparison between the full imaging dosimetry protocol (using 5 time points and 10 vertebrae) and the simplified protocol (using 2 time points and 3 vertebrae), again having all except one cycle within the 95% limits of agreement.

### Comparison

See Table 1.

**Table 1:** Bone marrow absorbed dose (mGy/GBq) for the 14 therapy cycles that were evaluated in this study

Cycle	Blood based dosimetry		Imaging based—5 time points, 8–10 vertebrae (T8–L5)		Imaging based—5 time points, 3 vertebrae (L3–L5)		Imaging based—2 time points, 8–10 vertebrae (T8–L5)		Imaging based—2 time points, 3 vertebrae (L3–L5)	
	D (mGy/GBq)	u(D) (%)	D (mGy/GBq)	u(D) (%)	D (mGy/GBq)	u(D) (%)	D (mGy/GBq)	u(D) (%)	D (mGy/GBq)	u(D) (%)
1	11.7	19.4	14.4*	18.5	13.3^$^	20.5	16.6*	26.7	13.8^$^	27.8
2	15.3	24.2	12.4	20.4	12.2	24.9	14.7	26.7	12.8	27.8
3	13.6	28.3	26.4	18.7	22.4	26.4	25.6	18.8	19.2	26.4
4	20.1	24.2	35.5	11.0	13.4	16.5	37.4	26.7	12.3	27.8
5	25.5	14.9	29.1*	19.7	15.7	19.6	32.7*	18.8	23.2	26.4
6	16.4	38.4	19.5	11.6	14.9	15.9	19.7	18.8	13.6	26.4
7	20.0	23.3	20.5	15.1	14.7	20.0	20.7	18.8	17.3	26.4
8	18.3	47.2	25.9^#^	18.0	17.0	19.1	25.9^#^	18.8	15.3	26.4
9	17.9	35.1	23.9*	20.2	24.1	17.8	26.0*	18.8	22.7	26.4
10	18.8	33.5	44.2^#^	15.3	48.1	14.2	42.7^#^	26.7	46.9	27.8
11	14.7	54.2	34.9	18.2	30.9	17.0	33.7	26.7	13.6	27.8
12	14.2	19.8	27.8*	18.1	27.5	21.0	35.1*	26.7	28.9	27.8
13	16.3	37.5	23.8*	18.9	17.9	21.9	24.4*	26.7	16.3	27.8
14	17.6	34.8	17.5^#^	15.6	13.1^$^	4.5	20.2^#^	26.7	14.0^$^	27.8
Mean ± SD	17.2 ± 3.4	31.1 ± 10.7	25.4 ± 8.7	17.1 ± 3.0	20.4 ± 9.9	18.5 ± 5.2	26.8 ± 8.4	23.3 ± 4.1	20.6 ± 9.7	27.2 ± 0.7

*9 vertebrae could be used

^#^8 vertebrae could be used

^$^2 vertebrae could be used

5 time points: 1 h, 1, 2, 3, 7 days; 2 time points: 1 and 7 days

## Discussion

This study investigated the possibility to use imaging data for bone marrow dosimetry after [^177^Lu]Lu-PSMA-617, and compared the absorbed dose outcomes with those determined from blood plasma measurements. It was shown that using post-treatment SPECT/CT images of the lower thoracic and lumbar vertebrae could indeed be used to calculate the bone marrow absorbed dose. This imaging-based method generally yielded slightly higher absorbed dose estimates than blood-based dosimetry, with higher variation between patients. A possible explanation for this higher dose could be the contribution of specific binding of PSMA to bone (marrow) cells as was also recently found in [^177^Lu]Lu-DOTATATE treatment [28], which would be missed in the blood-based methodology. This is confirmed by the increasing ratio between SPECT/blood measured activity over time, as was shown in Fig. 3. However, it is important to stress that by translating the vertebrae activity (or even as in this study: a part of the vertebrae) to total bone marrow absorbed dose, a homogeneous uptake pattern is being assumed throughout the bone marrow, thereby neglecting differences in cellularity by for instance prior therapies and other factors.

The higher variation in bone marrow absorbed dose in the imaging-based protocol could possibly represent actual patient-specific variation, as was also suggested in previous studies on other therapeutic compounds [14, 15]. However, this study evaluated only 8 patients (14 treatment cycles), so more patients would be needed to support these results. Furthermore, in this patient population, none of the patients experienced haematological toxicity, as was shown in previous studies [7, 23] and can be seen in Additional file 1: Online Resource 4 for measurements of hemoglobin, white blood cell count and thrombocytes. Therefore, bone marrow dosimetry could not be correlated to toxicity outcomes in this study. Longer follow-up time is needed to associate identified variation in bone marrow dosimetry to future bone-marrow insufficiency.

An imaging-based protocol using five-time point SPECT images and 10 vertebrae is challenging for clinical implementation. Ten vertebrae are often not visible on one bed position SPECT/CT, and the need for a high number of scans puts a high burden on both clinic and patients. Therefore, it is clinically relevant to simplify the dosimetry protocol while still ensuring reliable dosimetry. It was shown that bone marrow dosimetry could be performed using fewer time points and vertebrae without significant increase of uncertainty. A practical simplification to a two-time point imaging protocol (1 day and 7 days) and using vertebrae L3–L5 is suggested, however other simplified protocols could also be possible. To decide on the most optimal time points and vertebrae, it is important to consider the possible specific binding effects measurable at later time points (Fig. 3), as well as the uneven activity distribution over the vertebrae Fig. 4) (besides possible logistical preferences). A possible explanation for the higher activity measured in T11–L3 could be the proximity of these vertebrae to the kidneys, which could contribute to the detected counts due to scatter and/or spill-out. However, it is also possible that the higher activity measured in these vertebrae is the result of actual higher uptake. It is known that red marrow is not evenly distributed over all vertebrae, with thoracic vertebrae representing about 16% of total red marrow in the body, and lumbar vertebrae about 12% [24]. Even between specific thoracic and lumbar vertebrae, there might be differences in uptake.

This study evaluated the use of SPECT imaging for bone marrow dosimetry in mHSPC patient with limited tumor burden. In patients with advanced metastasized disease, using imaging for dosimetry might be difficult since the presence of extensive skeletal disease may preclude the ability to accurately delineate non-involved bone marrow for analysis [29]. A second aspect is that high bone metastasis load might also contribute to bone marrow absorbed dose, while this is not considered using only non-affected areas for translation to total bone marrow absorbed dose. Further research is necessary to develop the most optimal imaging-based bone marrow dosimetry protocol in patients with extended bone lesions. For patients with limited bone lesion burden, the imaging-based dosimetry methodology presented in this study could provide a practical and potentially more accurate method to aid in personalized toxicity monitoring and treatment design.

## Conclusions

This study showed that bone marrow absorbed dose after [^177^Lu]Lu-PSMA-617 can be determined using an imaging-based method of the lower vertebrae, and simplified using 2 time points (1 and 7 days) and 3 vertebrae. An increasing absorbed dose ratio over time between the imaging-based method and blood-based method suggests that there might be specific bone marrow binding of [^177^Lu]Lu-PSMA-617, which would make the imaging-based method potentially more accurate than the blood-based method. More research is needed in patients that experience haematological toxicity to correlate bone marrow absorbed dose to bone marrow toxicity. The method presented in this study offers a practical, easy and low burden protocol to determine bone marrow absorbed dose in patients receiving ^177^Lu]Lu-PSMA-617 and can thereby aid to personalize patient treatment.

### Supplementary Information

**Additional file 1: Online Resource 1. **Study flowchart.** Online Resource 2. **Acquisition and reconstruction parameters of the imaging protocols.** Online Resource 3. **Uncertainty analysis flowchart.** Online Resource 4. **Blood measurements for blood and bone marrow toxicity.

## Data Availability

The datasets generated during and/or analyzed during the current study are available from the corresponding author on reasonable request.

## Abbreviations

${\tilde{\text{A}}}$: Time integrated activity
CPM: Counts per minute
CRPC: Castration resistant prostate cancer
CT: Computed tomography
EANM: European Association of Nuclear Medicine
EMA: European Medicines Agency
FDA: American Food and Drug Administration
FOV: Field of view
ICRP: International Commission of Radiological Protection
mHSPC: Metastatic hormone sensitive prostate cancer
MIRD: Medical internal radiation dosimetry
PET: Positron emission tomography
p.i.: Post injection
PSA: Prostate specific antigen
SPECT: Single photon emission computed tomography
VOI: Volume of interest
