# Supplementary material for: Bone marrow dosimetry in low volume mHSPC patients receiving Lu-177-PSMA therapy using SPECT/CT
Source: EJNMMI Phys. 2024 Apr 3;11:34. doi: 10.1186/s40658-024-00636-0 (PMC10991600; doi:10.1186/s40658-024-00636-0)
Supplement: Supplementary file 1 — Additional file 1: Online Resource 1. Study flowchart. Online Resource 2. Acquisition and reconstruction parameters of the imaging protocols. Online Resource 3. Uncertainty analysis flowchart. Online Resource 4. Blood measurements for blood and bone marrow toxicity. [file 40658_2024_636_MOESM1_ESM.docx]

# Supplementary materials

**Title: Bone marrow dosimetry in low volume mHSPC patients receiving Lu-177-PSMA therapy using SPECT/CT**

**Authors:** Dagmar Grob^1^, Bastiaan M. Privé^1,2^, Constantijn H.J. Muselaers^4^, Niven Mehra^5^, James Nagarajah^1^, Mark W. Konijnenberg^1,3^, Steffie M.B. Peters^1^

^1^: Department of Medical Imaging, Radboud university medical center, Nijmegen, The Netherlands

^2^: Department of Radiation Oncology, Erasmus Medical Center, Rotterdam, The Netherlands

^3^: Department of Radiology and Nuclear Medicine, Erasmus Medical Center, Rotterdam, The Netherlands

^4^: Department of Urology, Radboud university medical center, Nijmegen, The Netherlands

^5^: Department of Medical Oncology, Radboud university medical center, Nijmegen, The Netherlands

**Corresponding author:**

Steffie M.B. Peters (ORCID: 0000-0002-0752-7134)

Radboud university medical center

Department of Medical Imaging

P.O. Box 9101

6500 HB Nijmegen, The Netherlands

T: +31 (6) 11621752

E: [steffie.peters@radboudumc.nl](mailto:steffie.peters@radboudumc.nl)


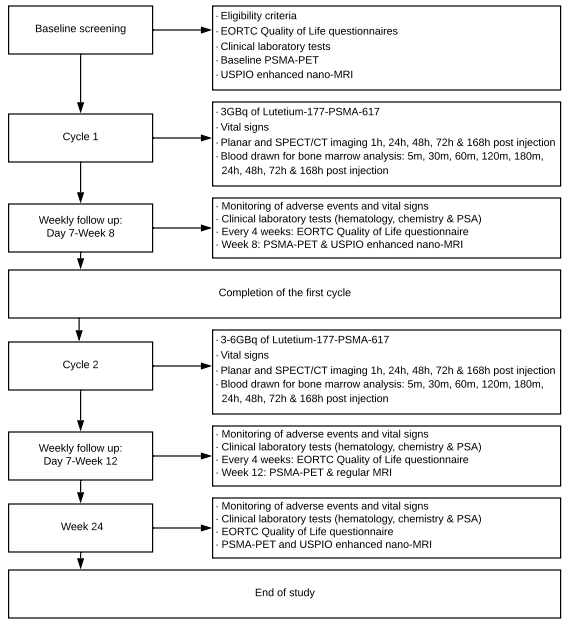


**Online Resource 1:** Study flowchart

**Online Resource 2: Acquisition and reconstruction parameters of the imaging protocols**

SPECT/CT and planar imaging was performed at 1, 24, 48, 72 and 168 hours after administration of [^177^Lu]Lu-PSMA on either a Symbia T16 or Symbia Intevo Bold system (Siemens Healthineers, Erlangen, Germany). SPECT/CT scans were acquired at three body regions: the pelvis, abdomen, and head-neck region (64 projections per detector, time per projection of 14 s, a 20% photon energy window at 208 keV, and dual-energy window for Compton scattering. SPECT data were reconstructed using OSEM reconstruction (Flash 3D with collimator detector response) using 4 iterations and 8 subsets, matrix size of 128 (resulting in a cuboid-shape voxel size of 4.8 mm^3^) and a smoothing Gaussian filter of 8.4 mm. The estimated reconstructed SPECT spatial resolution was 15 mm (FWHM). A low dose CT was performed (average DLP of 130 mGy·cm ) and the data were reconstructed using B31s kernel and 3.0 mm slice thickness resulting in a voxel size of 1.0 x 1.0 x 3.0 mm^3^. Of note, the SPECT image reconstruction approach takes into account corrections for scatter, CT-based attenuation, and dead-time.

**Online Resource 3: Uncertainty analysis flowchart**

1. Error in SPECT camera specific calibration factor for ^177^Lu: 5% [Peters, 2020]

CF = 10.6 ± 0.5 cps/MBq low count statistics at late time points was accounted for by Poisson distribution.

1. Data collection:
   1. Quantitative SPECT at 5 time-points, within 45 minute scan-time
   2. Activity concentration in blood samples at 9 time points; error: 5%
2. Drawing of VOIs in SPECT data over organs and tumor lesions to determine counts:
   1. Fixed size spherical VOI over central region of vertebrae; error: 10%
   2. ~~CT based VOI over organs; error 5%~~
3. Determination of lesion and organ volume:
   1. Lesion volume based on PET/CT and diagnostic CT [Jentzen, 2015];

Voxelisation and resolution error volume with lesion diameter d and voxel size a: $\left[ \frac{\delta V}{V} \right]^{2}=9 \left[ \frac{\delta d}{d} \right]^{2}=1.5\left( \frac{a}{d} \right)^{2}$ [Gear, 2018]

- 1. Organ volumes set at fixed ICRP-89 male phantom values; error 10%
  2. Bone marrow volume relative to red marrow content in vertebrae and total volume, systematic uncertainty in patients due to age, health status and prior therapy; e.g. 16% difference between S-values by Olinda (with 1170 g0 and IDAC-dose (1394 g), hence a 20% uncertainty in the S-value.

1. Fit to Time-Activity Curve
   1. Mono-exponential fit SPECT TAC when R^2^ > 0.7, determine covariance matrix
   2. Actual SPECT when R^2^ < 0.7 with exponential decay from last time-point
   3. Three-exponential curve fit of blood data, determine covariance matrix
2. Integration over time of the TAC, to determine the time-integrated activity Ã
   1. Mono-exponential integration, error in TIA or Ã:
      $\left[ \frac{\delta\tilde{A}}{\tilde{A}} \right]^{2}=\left[ \frac{\delta A_{0}}{A_{0}} \right]^{2}+\left[ \frac{\delta\lambda_{eff}}{\lambda_{eff}} \right]^{2}-\frac{2 Cov\left( A_{0},\lambda_{eff} \right)}{A_{0}\lambda_{eff}}$
   2. Trapezoid integration method; error:
      $\left[ \frac{\delta\tilde{A}}{\tilde{A}} \right]^{2}=\sum_{i} \left[ \frac{\delta A(t_{i})}{A(t_{i})} \right]^{2}$
   3. Three-exponential integration of blood concentration TAC, error in TIA or Ã:

$$\left[ \frac{\delta\tilde{A}}{\tilde{A}} \right]^{2}=\left[ \frac{\delta A_{0}}{A_{0}} \right]^{2}+{\left[ \frac{\delta P}{P} \right]^{2}+\left[ \frac{\delta\%Fast}{\%Fast} \right]^{2}+\left[ \frac{\delta\lambda_{Fast}}{\lambda_{Fast}+\lambda_{p}} \right]}^{2}+\left[ \frac{\delta\%Slow}{\%Slow} \right]^{2}+\left[ \frac{\delta\lambda_{Slow}}{\lambda_{Slow}+\lambda_{p}} \right]+\left[ \frac{\delta\lambda_{Medium}}{\lambda_{Medium}+\lambda_{p}} \right]-\sum covariances$$

3. Absorbed dose calculation with MIRD equation $D=\tilde{A}\times S$; error:

$$\left[ \frac{\delta D}{D} \right]^{2}={\left[ \frac{\delta\tilde{A}}{\tilde{A}} \right]^{2}+\left[ \frac{\delta S}{S} \right]}^{2}$$

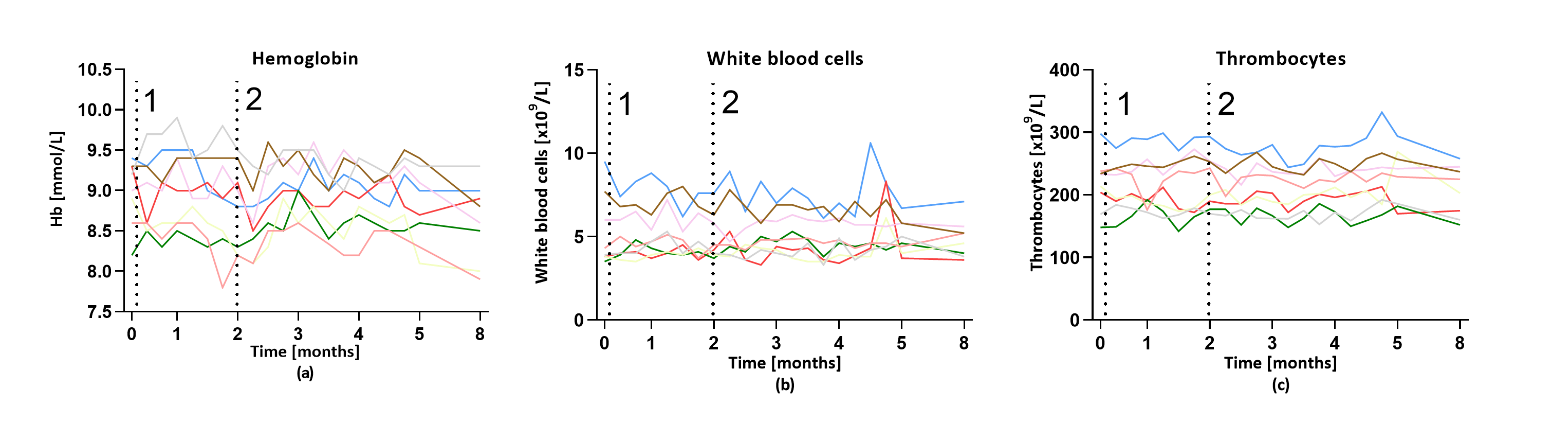


**Online Resource 4:** Blood measurements for blood and bone marrow toxicity. Each coloured line represents a patient. Dotted vertical lines indicate the first and second therapeutic cycle with ^177^Lu-PSMA-617. (a): hemoglobin, (b): white blood cells and (c): thrombocytes
